# Tumour purity assessment with deep learning in colorectal cancer and impact on molecular analysis

**DOI:** 10.1002/path.6376

**Published:** 2024-12-22

**Authors:** Lydia A Schoenpflug, Aikaterini Chatzipli, Korsuk Sirinukunwattana, Susan Richman, Andrew Blake, James Robineau, Kirsten D Mertz, Clare Verrill, Simon J Leedham, Claire Hardy, Celina Whalley, Keara Redmond, Philip Dunne, Steven Walker, Andrew D Beggs, Ultan McDermott, Graeme I Murray, Leslie M Samuel, Matthew Seymour, Ian Tomlinson, Philip Quirke, S:CORT consortium, Richard Adams, Michael Youdell, Viktor Koelzer, Simon Bach, Andrew Beggs, Celina Whalley, Louise Brown, Francesca Buffa, Peter Campbell, Jean‐Baptiste Cazier, Enric Domingo, Andrew Blake, Chieh‐His Wu, Aikaterini Chatzipli, Claire Hardy, Susan Richman, Philip Dunne, Keara Redmond, Paul Harkin, Steven Walker, Geoff Higgins, Jim Hill, Chris Holmes, Denis Horgan, Rick Kaplan, Richard Kennedy, Mark Lawler, Simon Leedham, Tim Maughan, Ultan McDermott, Gillies McKenna, Gary Middleton, Dion Morton, Graeme Murray, Phil Quirke, Sanjay Rathee, James Robineau, Manuel Salto‐Tellez, Les Samuel, Anna Schuh, David Sebag‐Montefiore, Matt Seymour, Ricky Sharma, Richard Sullivan, Ian Tomlinson, Nicholas West, Richard Wilson, Jens Rittscher, Tim Maughan, Enric Domingo, Viktor H Koelzer

**Affiliations:** ^1^ Department of Pathology and Molecular Pathology University Hospital and University of Zurich Zurich Switzerland; ^2^ Wellcome Sanger Institute Hinxton UK; ^3^ Institute of Biomedical Engineering (IBME), Department of Engineering Science, Old Road Campus Research Building University of Oxford Oxford UK; ^4^ Li Ka Shing Centre for Health Information and Discovery Big Data Institute, University of Oxford Oxford UK; ^5^ Oxford NIHR Biomedical Research Centre Oxford University Hospitals Trust Oxford UK; ^6^ Ground Truth Labs Ltd Oxford UK; ^7^ Department of Pathology and Tumour Biology Leeds Institute of Cancer and Pathology Leeds UK; ^8^ Department of Oncology University of Oxford Oxford UK; ^9^ Cantonal Hospital Baselland Institute of Pathology Liestal Switzerland; ^10^ Institute of Medical Genetics and Pathology University Hospital Basel Basel Switzerland; ^11^ Department of Cellular Pathology Oxford University Hospitals NHS Foundation Trust Oxford UK; ^12^ Nuffield Department of Surgical Sciences and NIHR Oxford Biomedical Research Centre University of Oxford Oxford UK; ^13^ Gastrointestinal Stem‐cell Biology Laboratory, Oxford Centre for Cancer Gene Research, Wellcome Trust Centre for Human Genetics University of Oxford Oxford UK; ^14^ Translational Gastroenterology Unit, Experimental Medicine Division, Nuffield Department of Clinical Medicine John Radcliffe Hospital Oxford UK; ^15^ Institute of Cancer and Genomic Science University of Birmingham Birmingham UK; ^16^ The Patrick G Johnston Centre for Cancer Research Queens University Belfast UK; ^17^ Almac Diagnostics Craigavon UK; ^18^ Department of Pathology, School of Medicine, Medical Sciences and Nutrition University of Aberdeen Aberdeen UK; ^19^ Department of Clinical Oncology Aberdeen Royal Infirmary, NHS GRAMPIAN Aberdeen UK; ^20^ University of Edinburgh Edinburgh UK; ^21^ Nuffield Department of Medicine Ludwig Institute for Cancer Research, University of Oxford Oxford UK; ^22^ University of Liverpool Liverpool UK; ^23^ Nuffield Department of Medicine University of Oxford Oxford UK

**Keywords:** pathology, artificial intelligence, colorectal cancer, diagnostic molecular pathology, personalised medicine

## Abstract

Tumour content plays a pivotal role in directing the bioinformatic analysis of molecular profiles such as copy number variation (CNV). In clinical application, tumour purity estimation (TPE) is achieved either through visual pathological review [conventional pathology (CP)] or the deconvolution of molecular data. While CP provides a direct measurement, it demonstrates modest reproducibility and lacks standardisation. Conversely, deconvolution methods offer an indirect assessment with uncertain accuracy, underscoring the necessity for innovative approaches. SoftCTM is an open‐source, multiorgan deep‐learning (DL) model for the detection of tumour and non‐tumour cells in H&E‐stained slides, developed within the Overlapped Cell on Tissue Dataset for Histopathology (OCELOT) Challenge 2023. Here, using three large multicentre colorectal cancer (CRC) cohorts (*N* = 1,097 patients) with digital pathology and multi‐omic data, we compare the utility and accuracy of TPE with SoftCTM versus CP and bioinformatic deconvolution methods (RNA expression, DNA methylation) for downstream molecular analysis, including CNV profiling. SoftCTM showed technical repeatability when applied twice on the same slide (*r* = 1.0) and excellent correlations in paired H&E slides (*r* > 0.9). TPEs profiled by SoftCTM correlated highly with RNA expression (*r* = 0.59) and DNA methylation (*r* = 0.40), while TPEs by CP showed a lower correlation with RNA expression (*r* = 0.41) and DNA methylation (*r* = 0.29). We show that CP and deconvolution methods respectively underestimate and overestimate tumour content compared to SoftCTM, resulting in 6–13% differing CNV calls. In summary, TPE with SoftCTM enables reproducibility, automation, and standardisation at single‐cell resolution. SoftCTM estimates (*M* = 58.9%, SD ±16.3%) reconcile the overestimation by molecular data extrapolation (RNA expression: *M* = 79.2%, SD ±10.5, DNA methylation: *M* = 62.7%, SD ±11.8%) and underestimation by CP (*M* = 35.9%, SD ±13.1%), providing a more reliable middle ground. A fully integrated computational pathology solution could therefore be used to improve downstream molecular analyses for research and clinics. © 2024 The Author(s). *The Journal of Pathology* published by John Wiley & Sons Ltd on behalf of The Pathological Society of Great Britain and Ireland.

## Introduction

The assessment of the cancer microenvironment plays a pivotal role in informing the interpretation of transcriptional signatures and copy number variation (CNV) calls within molecular pathology workflows [1, 2]. Semi‐quantitative visual evaluations of tumour purity (TP) are commonly employed to gauge sample adequacy prior to omic profiling. However, these estimations suffer from a lack of standardisation and exhibit poor reproducibility, potentially introducing bias into genomic analyses [3, 4]. Bioinformatic deconvolution techniques for estimating tumour content from genomic data offer a potential solution but are costly to implement, lack spatial preservation, and demonstrate relevant failure rates [5, 6, 7]. In contrast, computational pathology (CPATH) has emerged as a robust and non‐destructive approach to identifying and segmenting neoplastic and non‐neoplastic cell populations within clinical pathology samples, seamlessly integrating into existing laboratory workflows. In this study, we explored the potential of CPATH to enhance diagnostic molecular pathology, comparing it directly with expert pathologist assessment and established deconvolution methods using transcriptional and DNA methylation data. The findings of this investigation carry significant implications for selecting the most suitable method for both clinical and research purposes. Given the importance of TP estimations (TPEs) in interpreting CNV calls and next‐generation sequencing (NGS) readouts in molecular pathology, we postulate that a more precise approach to cell deconvolution could greatly benefit colorectal cancer (CRC) molecular pathology workflows and cancer genomics in general.

DNA aneuploidy has been associated with a shorter disease‐free and overall survival in CRC patients [8, 9]. NGS data can be utilised to derive information on copy number variations following TPE from deconvolution methods. However, the accuracy of this approach remains uncertain due to the lack of correlation with conventional pathology (CP) TPEs [4, 10]. Conventional methods to measure CNVs involve tissue dissociation and assessment of DNA content using flow cytometry or microscopic imaging [9]. Since copy number alterations are diluted by an increase in normal diploid cells, accurately determining TP is crucial for bioinformatic correction of CNV calls. By extracting precise measures of TP, CPATH has the potential to improve the identification of CRC cases harbouring aneuploid neoplastic populations.

Here we perform a comprehensive analysis of TP in three cohorts with a total of 1,097 CRC patients from the Stratification in COloRecTal cancer (S:CORT) programme and The Cancer Genome Atlas (TCGA) with full molecular information and available whole slide images (WSIs). We compare the impact and utility of (1) a gold‐standard molecular deconvolution method (ESTIMATE) with (2) CP, (3) a DNA‐methylation‐based deconvolution method (InfiniumPurify), and (4) a novel open‐source, multiorgan CPATH algorithm (SoftCTM) on TPEs, including downstream CNV analysis and biological stratification.

Our systematic analysis reveals that SoftCTM‐based TPE surpasses the accuracy of CP, ESTIMATE, and InfiniumPurify. These currently established methods tend to respectively underestimate and overestimate TP. TPE using SoftCTM offers analytical robustness, automation, and standardisation, resulting in remarkably high reproducibility at the single‐cell level. Leveraging CPATH approaches could therefore enhance the planning and evaluation of subsequent molecular analyses.

## Materials and methods

### Cohorts

**Figure 1:**
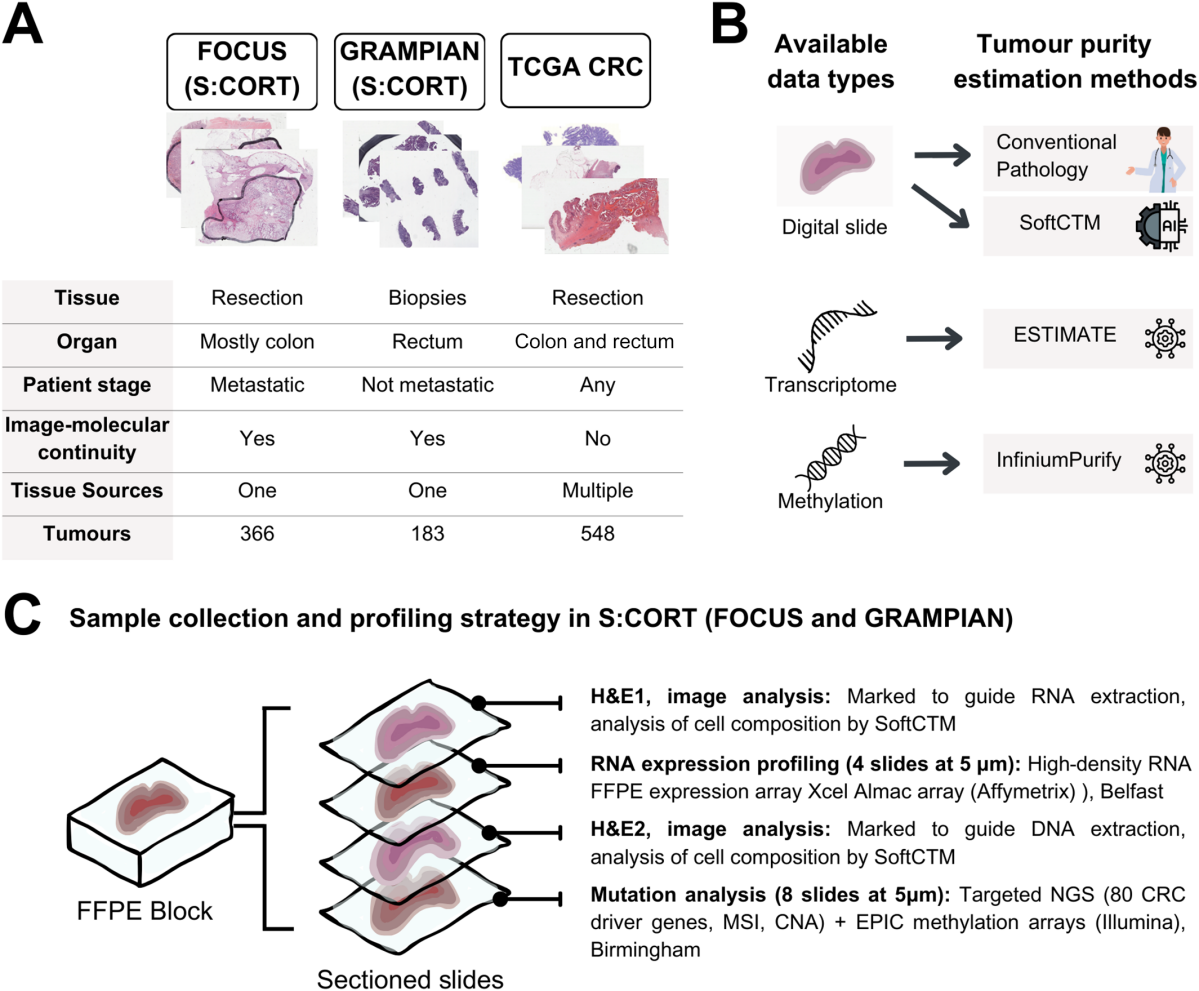
Experimental study design. (A) Specifications and data summary of the three independent datasets (FOCUS, TCGA, and GRAMPIAN) used in this study. (B) Available data types and tumour estimation methods applied on each data type. (C) Sample collection and profiling strategy in FOCUS and GRAMPIAN cohorts. Created with canva.com.

This study included a total of 1,097 CRC cases from three independent cohorts (FOCUS, GRAMPIAN, TCGA) with complete digital pathology (DP) and multi‐omic datasets. Cases from FOCUS and GRAMPIAN were characterised as part of the Medical Research Council (MRC) Cancer Research UK (CRUK) S:CORT programme, the S:CORT case IDs considered in this study are detailed in supplementary material, Table S1. Figure 1A provides an overview of the three datasets' specifications, Figure 1B indicates the available data types and applied TPE methods, and Figure 1C shows their sample collection strategy. Further details on data and resource availability can be found in supplementary material, Figure S1.

#### Cohort 1: FOCUS

As part of the Stratification in the S:CORT programme, 385 patients with available formalin‐fixed paraffin embedded (FFPE) blocks of the primary CRC were selected from the MRC FOCUS randomised clinical trial (RCT) that tested different strategies of sequential and combination chemotherapy for patients with advanced CRC [11]. Serial sections were cut from one representative block for H&E staining followed by four unstained sections for RNA extraction, a second H&E‐stained section, and eight unstained sections for DNA extraction (Figure 1). Glass H&E slides were re‐reviewed by an expert gastrointestinal pathologist and tumour tissue with the associated intra‐tumoural stroma was annotated in the first and second H&E‐stained section respectively to guide RNA and DNA extractions. No tumour microdissection was performed. Regions of extensive necrosis and non‐tumour tissue were excluded according to standard practice for downstream molecular tumour profiling. RNA expression microarrays (Xcel array, Affymetrix, Santa Clara, CA, USA), DNA target capture (SureSelect, Agilent, Santa Clara, CA, USA) followed by NGS sequencing (Illumina, San Diego, CA, USA), and DNA methylation arrays (EPIC arrays, Illumina) were applied in that order [12]. All H&E slides were scanned on an Aperio scanner at 20×. Digital slides were re‐reviewed by a second gastrointestinal pathologist and tumour region annotations for deep‐learning (DL) classification were generated. Areas containing folds or debris were excluded by digital annotation. Samples were excluded if they contained biopsies instead of resections and/or metastatic tissue instead of primary lesions or extensive ulceration/necrosis. Slides with irrecoverable failure of the staining or scanning procedure were excluded for technical reasons. The final set was composed of 702 primary tumour H&E slides from 366 cases.

#### Cohort 2: TCGA

A total of 624 digital slides from 615 cases of colon and rectal adenocarcinoma were downloaded from the TCGA Data Portal (COAD and READ datasets, https://www.cancer.gov/tcga, last accessed: 2 August 2018). All digital slides were re‐reviewed, and tumour tissue was annotated. Slides were excluded based on the same quality control (QC) criteria as described for FOCUS. Gene‐level expression data were downloaded with the R package TCGAbiolinks [13]. After excluding slides based on QC and six duplicated tumours, the final number of slides was 548, all from unique cases.

#### Cohort 3: GRAMPIAN

A total of 334 slides from 184 pretreatment biopsy FFPE blocks from rectal cancer patients were analysed for this study as part of the S:CORT programme [14, 15]. Following the initial biopsy, all patients received preoperative (chemo)radiotherapy followed by surgical resection. Slides and molecular profiling were processed as described for cohort 1 (FOCUS) but using five to nine sections for RNA extraction and nine for DNA. A total of four slides were excluded after QC for a final set of 332 slides from 183 cases.

### Ethics approval

The FOCUS and GRAMPIAN cohorts of S:CORT have ethical approval (REC 15/EE/0241) from the East of England – Cambridge South Research Ethics Committee. TCGA (https://www.cancer.gov/tcga, last accessed: 2 August 2018) is an open‐source public database.

### Analysis of cell composition by deep learning

**Figure 2:**
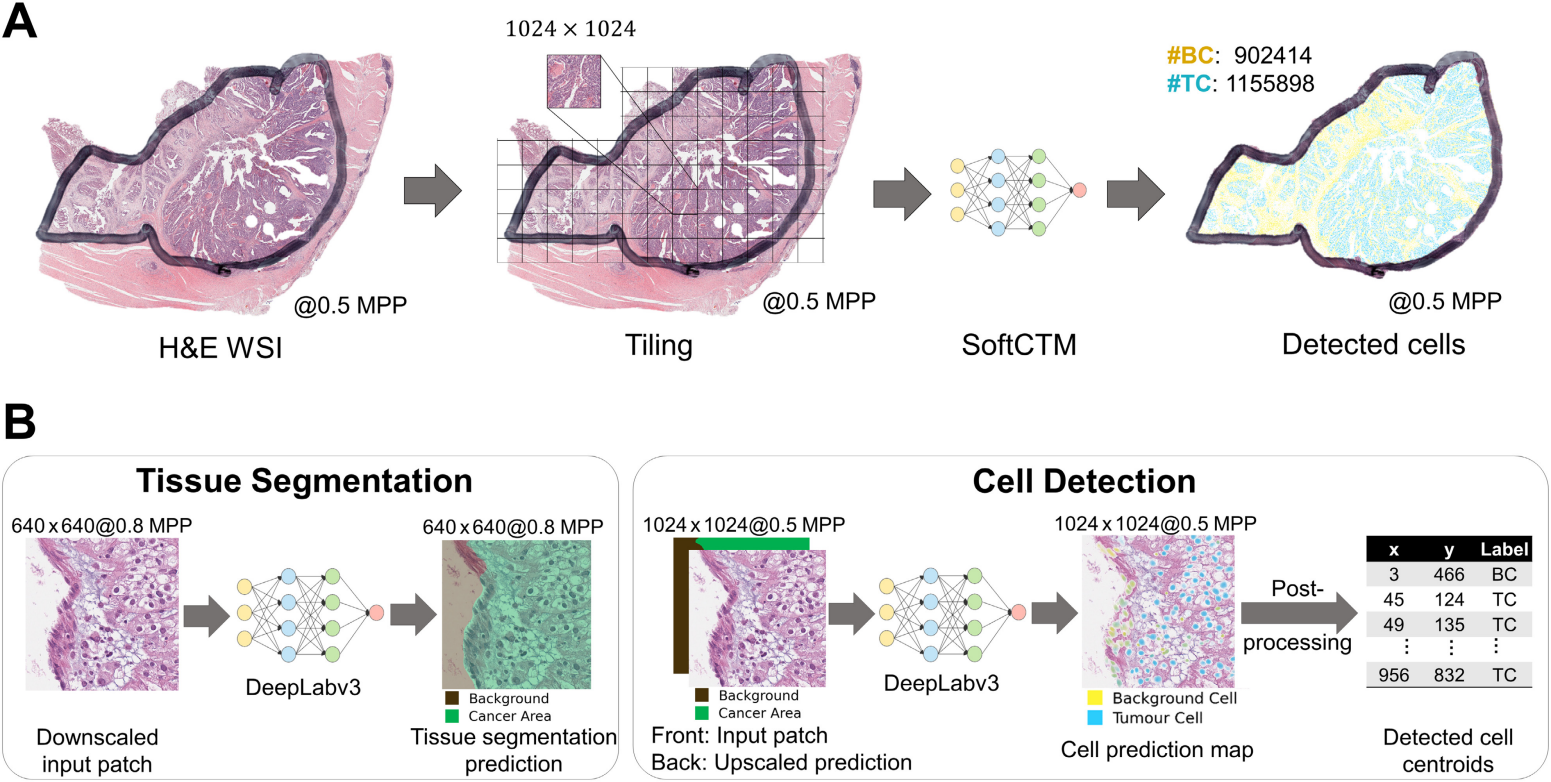
Inference workflow for tumour and background cell nuclei (TC, BC) detection by SoftCTM on a H&E‐stained WSI. (A) WSI‐level inference: Pathologist‐marked ROIs of a H&E‐stained WSI are tiled into patches (1,024 × 1,024 pixels at 0.5 MPP), on which SoftCTM is applied. The predictions are then recombined into a spatially resolved WSI‐level prediction of detected TC and BC. (B) Patch‐level inference: The SoftCTM algorithm consists of two stages: tissue segmentation and cell detection. Tissue segmentation is performed at 0.8 MPP, cell detection at 0.5 MPP. For cell detection, the tissue segmentation prediction is used as input along with the input patch. The output is a probability map for each cell class, from which detected cells are extracted through a postprocessing step. MPP, microns per pixel; WSI, whole slide image

We utilised the Soft Cell‐Tissue DL model (SoftCTM) [16] to detect tumour and non‐tumour cells in the test cohorts. The model was developed on the Overlapped Cell on Tissue Dataset for Histopathology (OCELOT) training and validation set [17] within the OCELOT 2023 Challenge. The sets respectively comprised *n* = 400 and *n* = 137 pairs of annotated cell patches at 50× and tissue patches at 12.5× extracted from 173 and 65 TCGA slides and six distinct organs (bladder, endometrium, head and neck, kidney, prostate, and stomach). SoftCTM consists of a model for tissue segmentation and cell detection (Figure 2). The tissue segmentation prediction was provided as input to the cell detection model, allowing consideration of predicted tumour versus non‐tumour tissue regions. As our test cohort slides were at 20×, we utilised the SoftCTM 20× version and WSI inference pipeline from the public GitHub repository (https://github.com/lely475/ocelot23algo, last accessed: 27 May 2024). Contrary to [16], the WSI inference pipeline does not extract a larger field of view for tissue segmentation or apply test‐time augmentation for either model, thereby reducing computational costs. We inferred the SoftCTM model on each slide (Figure 2A) and collected the tumour and background (non‐tumour) cell counts (TC, BC) (Figure 2B). Visualisations of SoftCTM predictions (supplementary material, Figure S1) were found to be of high quality based on visual review by an experienced board‐certified pathologist (VHK).

### Correlation of SoftCTM with pathologist‐supervised DP algorithm

To further verify the reliability of the SoftCTM algorithm, we investigated its correlation with a CPATH algorithm for cell detection, developed within the Indica Labs HALO AI™ digital image analysis platform [18]. Further referred to as HALO DP, the algorithm consisted of (1) a tissue segmentation algorithm, which was trained on >1,500 tissue areas from S:CORT, TCGA, TEM, and CORGI CRC cohorts with pathologist annotations (tumour, desmoplastic stroma, inflamed stroma, muscle, necrosis, mucin, mesenchyme, background) and (2) a visually optimised general cell segmentation algorithm implemented in the HALO image analysis platform. Algorithms 1 and 2 were combined for cell classification, where all cells within a predicted tissue area were treated as the respective tissue's cells (e.g. all cells in the tumour area are treated as tumour cells). This provides a more fine‐grained distinction between different non‐neoplastic cell classes than SoftCTM, but it is more restricted in the assumption that a specific tissue area cannot contain other cell types. As with SoftCTM, we derived TPEs for HALO DP as tumour cell counts divided by total cell counts. TPEs by SoftCTM and HALO DP showed a high correlation of 0.78 considering all cohorts (supplementary material, Figure S3), with a Pearson correlation coefficient of 0.85 and 0.79 for FOCUS and GRAMPIAN cohorts and slightly lower, 0.68, for TCGA.

### Preprocessing of image data and exclusion criteria

Digital slides were re‐reviewed and invasive cancer regions annotated by an experienced board‐certified pathologist (VHK) using the HALO™ software version 2.3.2089.52 (Indica Labs, Corrales, NM, USA). The SoftCTM algorithm was applied within the annotated regions.

### Tumour purity estimations

All TPEs were harmonised to scale 0% (e.g. no tumour) to 100% (pure tumour). CPATH estimations were determined using cell counts by SoftCTM (tumour cell counts divided by total cell counts). CP estimations were derived in TCGA and GRAMPIAN by one expert pathologist visually estimating the proportion of viable tumour versus all cells at fractions of 5%. In FOCUS, scores from two different pathologists were available showing modest correlation [*r* = 0.59, CI = (0.52, 0.65)]. One of them was randomly selected for all further analyses. Estimations from multi‐omic data were derived from transcriptome with ESTIMATE [19] and from methylation with InfiniumPurify [20] using their original R packages in FOCUS and GRAMPIAN. For TCGA, purity based on ESTIMATE and InfiniumPurify were retrieved from previous publications [10, 21].

### Gene copy number estimation

The targeted NGS panel applied to FOCUS and GRAMPIAN cohorts contains probes spanning SNPs evenly distributed along the human genome (average of one SNP per 3 Mb) and 66 chromosomal regions recurrently gained or lost in CRC. This design allows for the generation of copy number estimations from targeted NGS at row resolution, acknowledging that such estimations may not be directly comparable to techniques that examine the entire genome. Furthermore, CNVkit [22], a tool specifically designed to enhance targeted NGS data by the analysis of both targeted and off‐target reads, was used on both cohorts adjusting by TPEs from different methods. Copy number segments with estimations ≥3 were classified as gain, 2 as neutral, and ≤1 as loss. The Whole Genome Instability Index (WGII) measuring the proportion of the genome with an aberrant copy number was calculated as the sum of the lengths of calls for either loss or gain divided by the whole length.

### Consensus molecular subtype (CMS) classification

CMS was derived as described previously [14]. In brief, the R library CMSclassifier [23] was used to compute both single sample predictions after row‐centring the expression data and random forest in each of the three cohorts separately. CMS calls were generated by matching both methods without applying any cut‐off.

### Statistics

Correlations were analysed using Pearson's correlation coefficient, with confidence intervals provided at 95%. Statistical differences between TPE methods were evaluated using paired‐samples *t*‐tests. We used the SciPy [24] statistics Python package for Pearson correlation analysis, paired‐samples *t*‐test, generation of boxplots and histograms and Pingouin [25] for generating Bland–Altman plots.

## Results

### Assessment of cell composition by DL is accurate, robust, and reproducible

The objective of this study was to evaluate the efficacy of a reliable and openly accessible CPATH technique for estimating TP on CRC histology slides and to conduct a comprehensive comparison with CP and molecular deconvolution methods (ESTIMATE, InfiniumPurify). Test cohorts including a total of 1,097 patients were selected to represent relevant clinical scenarios in the management of CRC patients including postoperative resection specimens (FOCUS, *n* = 702 slides from 366 patients; TCGA, *n* = 548 slides from 548 patients) and endoscopic biopsy material (GRAMPIAN, *n* = 332 slides from 183 patients) (Figure 1A). Tumour areas on each slide were annotated by a pathologist, and molecular analysis was performed on material obtained from strict serial sections (FOCUS and GRAMPIAN). Please refer to Sirinukunwattana *et al* [14] (https://gut.bmj.com/content/gutjnl/70/3/544/DC2/embed/inline-supplementary-material-2.pdf, last accessed: 29 October 2024) for clinical and molecular data characterisation and a summary of the GRAMPIAN and FOCUS cohorts. Technical reproducibility was checked by rerunning SoftCTM on 50 H&E slides from FOCUS, comparing cell counts and TPEs in each paired output. The correlation was excellent with an *r*‐coefficient equalling 1.0, showing technical repeatability and stability of SoftCTM predictions. In contrast, CP estimates by two different pathologists in the FOCUS cohort showed only mild correlation [*r* = 0.59, CI = (0.52, 0.65)]. As SoftCTM was not trained on CRC histology, we further investigated its agreement with a pathologist‐supervised CPATH algorithm that was trained utilising parts of the test cohort data and report a high level of agreement in TPE between the methods (supplementary material, Figure S3). To investigate intra‐sample variance, we then compared TPE by SoftCTM between the first and second H&E slides in all available cases from FOCUS and GRAMPIAN (334 and 149 pairs respectively) (Table 1 and supplementary material, Figure S4). Importantly, these slides represent serial sections with approximately 20 μm *Z*‐axis distance for FOCUS and 40 μm for GRAMPIAN, with additional material taken for RNA profiling between the sectioning planes of interest. Across serial sections, both the TPE and total cell counts showed excellent correlations (all *r* > 0.8, Table 1). We further verified that the number of detected cells correlated with the size of the invasive cancer region [supplementary material, Figure S5, *r* = 0.925, CI = (0.916, 0.933)]. For this comparison, only tissue regions inside the expert pathologist annotation were considered. The mean cell density in resection specimens from the FOCUS and TCGA cohorts was approximately 19,000 cells/mm^2^, whereas GRAMPIAN biopsy specimens exhibited a cellular density of approximately 26,000 cells/mm^2^ indicating increased tissue compression in biopsy samples. This difference may be attributed to the sampling technique involving compression by biopsy forceps and enhanced fixation of smaller tissue samples, resulting in greater shrinkage.

**Table 1:** Pearson correlation of tumour purity estimation, tumour, and background cell counts by digital pathology for H&E sections 1 and 2, which gives insight into the reproducibility and dependency of the method on the selected tissue section (all *p* < 0.001).

	TPE	Tumour cells	Background cells
FOCUS (*N* = 334)	*r* = 0.949, CI = (0.937, 0.959)	*r* = 0.976, CI = (0.970, 0.981)	*r* = 0.908, CI = (0.887, 0.925)
GRAMPIAN (*N* = 149)	*r* = 0.818, CI = (0.757, 0.865)	*r* = 0.976, CI = (0.967, 0.982)	*r* = 0.946, CI = (0.927, 0.961)
Total (*N* = 485)	*r* = 0.916, CI = (0.901, 0.929)	*r* = 0.983, CI = (0.980, 0.989)	*r* = 0.945, CI = (0.934, 0.954)

Abbreviations: CI, confidence interval; *r*, Pearson correlation coefficient.

### Tumour purity assessed by different methods

**Figure 3:**
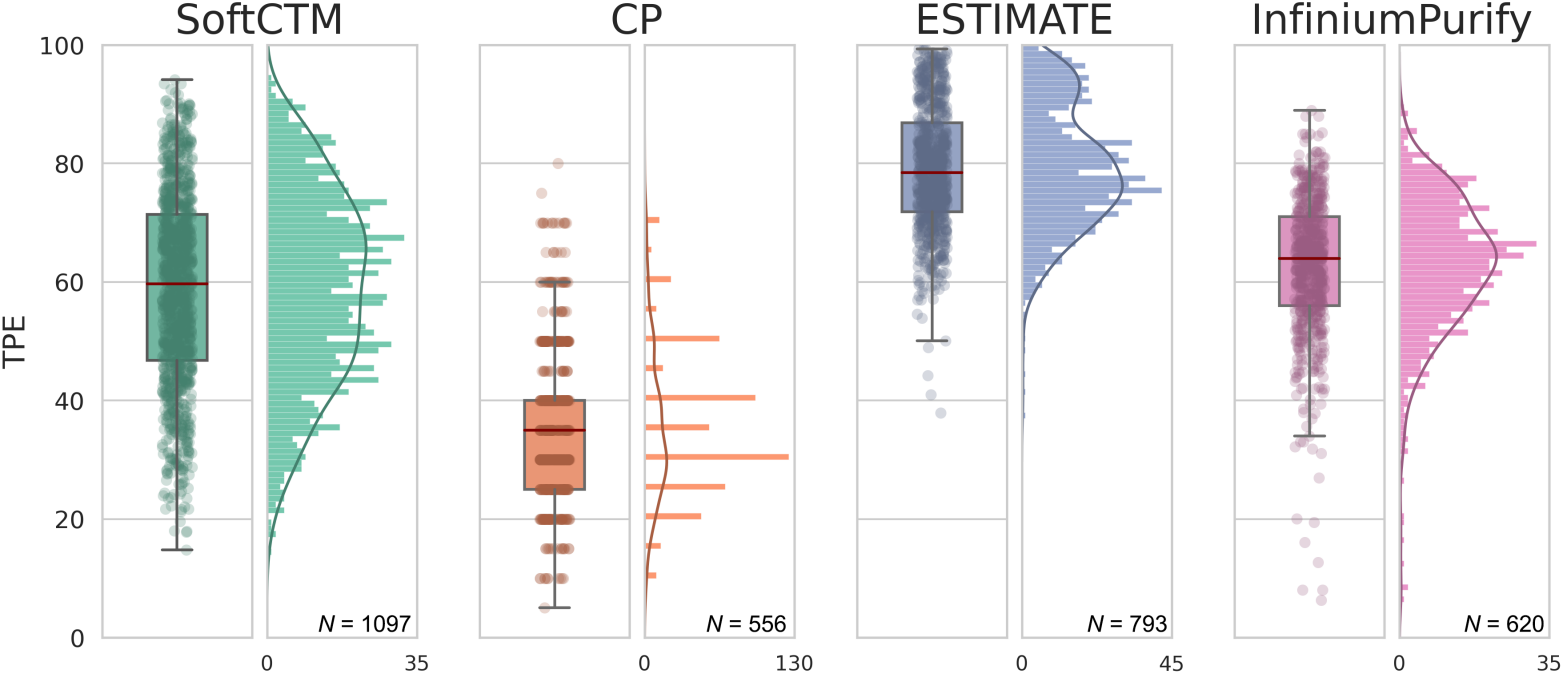
Boxplot and histogram comparing distribution of TP estimated by different methods for the combined test cohorts. We only consider samples with TPE available for all methods. The box represents the IQR, encompassing 50% of the data points. Red line indicates median, and whiskers extend to ±1.5 IQR from IQR edges. TP, tumour purity; IQR, interquartile range

Next, we compared TPEs derived from cell counts of SoftCTM with visual estimates determined by expert pathologists and TPEs derived from bioinformatic deconvolution from RNA expression using ESTIMATE [19] and DNA methylation using InfiniumPurify [20]. We first conducted a statistical comparison of all TPE methods using paired‐samples *t*‐tests, revealing significant differences between each method (*p* < 0.001) (supplementary material, Figure S6). SoftCTM displays a broad distribution of TPE predictions, aligning with biological expectations. In contrast, the other three methods demonstrate narrower distributions, and these converge at varying levels (Figure 3, and cohort‐specific in supplementary material, Figure S7). Our objective was to provide additional context to these findings by considering CRC biological subgroups. To achieve this, we employed the primary transcriptomic classifier CMS as a framework, given its significant association with TPE metrics. Notably, CMS1 and CMS4 subtypes are associated with elevated levels of immune and stromal infiltration, resulting in lower TPE, whereas the canonical and metabolic subgroups (CMS2 and CMS3) are distinguished by higher epithelial content [14, 23]. As expected, we observed lower TPEs in CMS4 followed by CMS1 and higher epithelial content in CMS2/3 (supplementary material, Figure S8). These results suggest that all four methods reliably capture the expected associations across biological subgroups.

**Figure 4:**
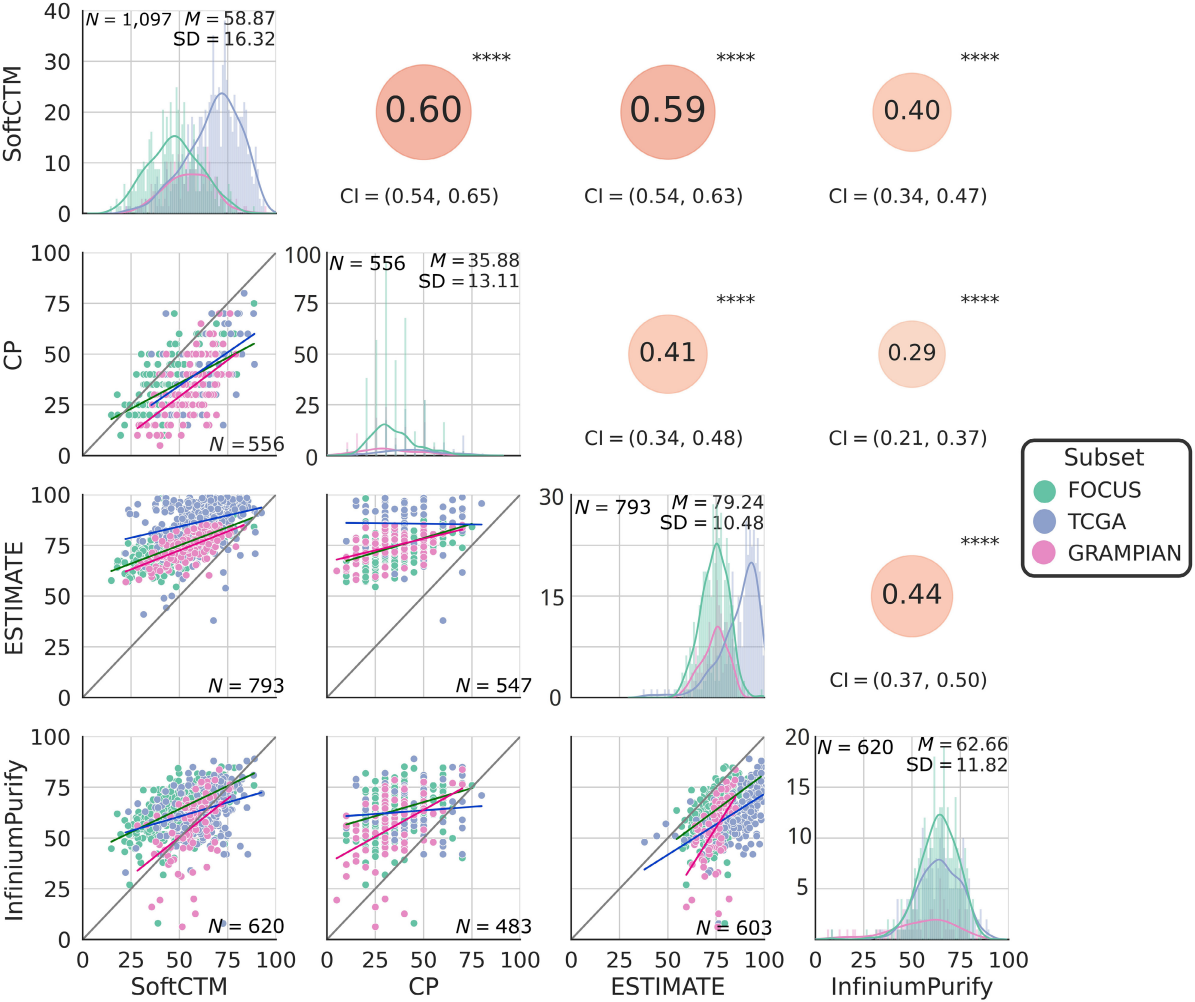
Comparison of TPE method results for test cohorts. Below diagonal: scatter plots comparing respective TPE method results. Diagonal: histogram for each TPE method with mean (M) and standard deviation (SD) in top right. Above diagonal: Pearson correlation coefficient between respective TPE methods; ****p < 0.0001. CI, confidence interval; TPE, tumour purity estimation.

We further examined the direct correlation between SoftCTM, CP, and molecular TPE methods (ESTIMATE, InfiniumPurify) (Figure 4, and cohort‐specific in supplementary material, Figure S9). The correlation coefficients (*r*) comparing SoftCTM with ESTIMATE and InfiniumPurify exhibited strong agreement in FOCUS and GRAMPIAN cohorts (all *r* in 0.49–0.70). Conversely, in TCGA, where spatial continuity between molecular and pathological profiles is lacking, the correlation was notably lower (all *r* in 0.29–0.32). The correlations of SoftCTM with CP were generally strong (all *r* in 0.53–0.61). Notably, tumour content tended to be overestimated when analysed by ESTIMATE and, to a lesser degree, by InfiniumPurify, compared to SoftCTM, while we observed underestimation by CP. When comparing CP with ESTIMATE and InfiniumPurify TPEs, we observed a mild correlation in FOCUS and GRAMPIAN (all *r* in 0.31–0.56) and note an overestimation by the molecular deconvolution methods compared to CP. In TCGA, there was no correlation between CP and molecular methods (all *r* ≈0.0).

### Copy number adjusted using TPEs from CP, DP, and bioinformatic deconvolution methods

**Figure 5:**
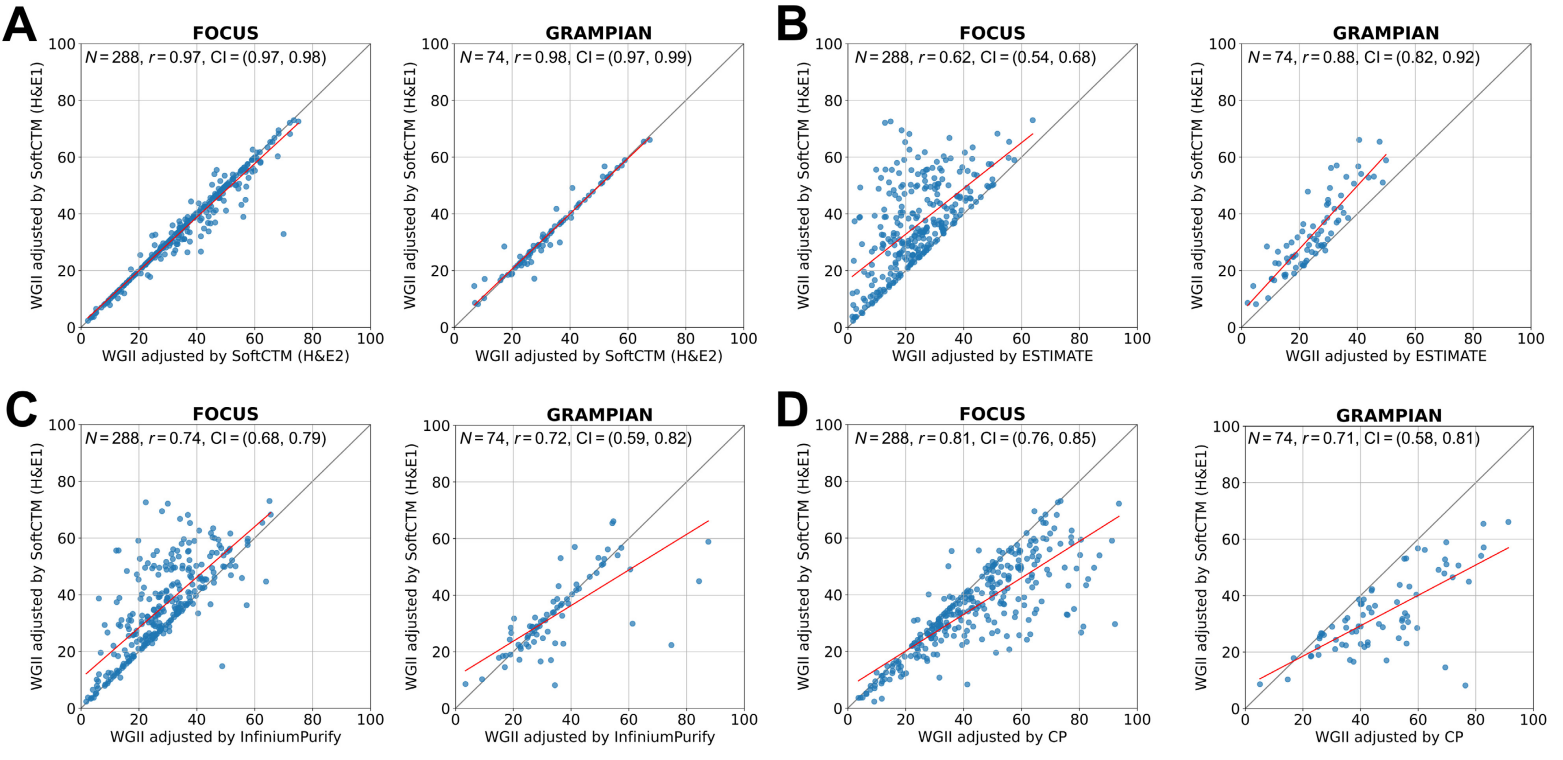
Comparison of Whole Genome Instability Index (WGII) adjusted by (A) SoftCTM, (B) ESTIMATE, (C) InfiniumPurify, and (D) CP; all *p* < 0.001. CI, confidence interval.

Our findings revealed variation in TP distribution depending on the assessment method. This variability can significantly impact the correction of downstream metrics in molecular pathology analysis. One common application involves using TPEs to normalise copy number data, which may be affected by increasing proportions of non‐tumour, diploid cells in DNA extractions. Here, we quantify the impact of utilising TPEs derived from different methods on copy number analysis. Specifically, we assessed the WGII, which measures the proportion of the genome that deviates from diploidy, within the FOCUS and GRAMPIAN cohorts (Figure 5). The TCGA cohort was excluded for this analysis, as we compared copy number adjustment by TPE of SoftCTM in H&E1 with H&E2, which is not feasible in TCGA with only a single H&E slide available.

The correlation of WGII following copy number adjustment using TPEs from SoftCTM for H&E1 and H&E2 was excellent [FOCUS: *r* = 0.973, CI = (0.966, 0.979), GRAMPIAN: *r* = 0.978, CI = (0.965, 0.986)]. In contrast, comparison of TPEs by SoftCTM with TPEs from RNA (ESTIMATE) and DNA methylation (InfiniumPurify) revealed lower correlations [FOCUS: *r* = 0.617, CI = (0.540, 0.683), GRAMPIAN: *r* = 0.881, CI = (0.818, 0.924) and FOCUS: *r* = 0.741, CI = (0.684, 0.789), GRAMPIAN: *r* = 0.724, CI = (0.594, 0.818)]. Compared to SoftCTM, ESTIMATE clearly underestimated WGII, while InfiniumPurify underestimated WGII for FOCUS but showed mixed patterns with a tendency towards overestimation for GRAMPIAN. Similarly, a lack of correlation was observed in comparisons of SoftCTM TPEs with CP [FOCUS: *r* = 0.809, CI = (0.765, 0.845), GRAMPIAN: *r* = 0.714, CI = (0.581, 0.811)], where CP tends to overestimate WGII.

We then measured changes in copy number at the chromosome arm level (Table 2). When comparing SoftCTM for H&E1 and H&E2, only ~1.5% of copy number segments differed between both cohorts, with a slight tendency towards overcalling in FOCUS (76.06%) and a balanced distribution in GRAMPIAN. However, following adjustment with TPEs from ESTIMATE, 9–13% of calls differed, nearly all of them being underestimations. Conversely, when compared to following adjustment with TPEs from CP, the difference was also 8–14%, but most of them were overestimations. For adjustment with InfiniumPurify TPEs we note 6–9% differing calls with primarily underestimations in FOCUS and a tendency (67.2%) to overcalling in GRAMPIAN.

**Table 2:** Comparison of copy number alteration calls in 39 chromosomal arms adjusted by different tumour purity estimation (TPE) methods for FOCUS (*N* = 288) and GRAMPIAN (*N* = 74): TPE from SoftCTM applied on H&E1, SoftCTM applied on H&E2, transcriptome‐based ESTIMATE, DNA‐methylation‐based InfiniumPurify, and conventional pathology. Undercalling refers to copy number alterations (CNAs, both losses and gains) that were detected by SoftCTM (H&E1), but not by the respective other method, and the opposite for overcalling.

	CNA calls in Chr arms		SoftCTM (H&E2)			Estimate			InfiniumPurify			CP		
			Loss	Neutral	Gain	Loss	Neutral	Gain	Loss	Neutral	Gain	Loss	Neutral	Gain
FOCUS	SoftCTM (H&E1)	Loss	2,066	23	1	1,340	749	1	1,622	466	2	2,034	55	1
		Neutral	72	6,662	74	0	6,808	0	19	6,761	28	416	6,008	384
		Gain	0	22	2,312	0	675	1,659	0	458	1,876	0	43	2,291
	Different calls		192/11,232 (1.7%)			1,425/11,232 (12.7%)			973/11,232 (8.7%)			899/11,232 (8.0%)		
	Undercalling		51/192 (23.4%)			1,424/1,425 (99.9%)			103/973 (95.0%)			98/899 (10.9%)		
	Overcalling		147/192 (76.6%)			1/1,425 (0.1%)			906/973 (5.0%)			801/899 (89.1%)		
GRAMPIAN	SoftCTM (H&E1)	Loss	474	15	0	363	126	0	456	33	0	489	0	0
		Neutral	6	1,865	8	0	1,878	1	56	1,760	63	197	1,455	227
		Gain	0	14	504	1	118	399	2	26	490	3	2	513
	Different calls		43/2,886 (1.5%)			246/2,886 (8.5%)			180/2,886 (6.2%)			429/2,886 (14.9%)		
	Undercalling		29/43 (67.4%)			244/246 (99.2%)			59/180 (32.8%)			2/429 (0.5%)		
	Overcalling		14/43 (32.6%)			2/246 (0.8%)			121/180 (67.2%)			427/429 (99.5%)		

## Discussion

H&E slides are routinely prepared in the work‐up of CRC tissue samples in pathology laboratories. TPE serves as an important QC metric for selecting tissue material suitable for molecular tumour profiling [26] and is essential for the correct interpretation of molecular diagnostic tests [27]. Here, we propose a DL‐based cell‐level TPE method and demonstrate its high reproducibility, correlation to other TPE methods, and implications for downstream molecular analyses on a large CRC dataset, including three separate cohorts with complete DP and multi‐omic data.

While the assessment of tumour percentage on a given tissue slide may appear to be a straightforward task, it is more complex than initial observation might suggest [28]. Interobserver reproducibility of CP between domain experts is low to moderate [4, 29]. Previous research associated this variability with insufficiently defined cellularity criteria and underestimation of the non‐linear correlations between area‐ and cell‐level assessments [30, 31]. This effect is particularly pronounced in cases with very high or low cellular density in regions of interest (ROIs) [30]. These limitations reflect negatively on the quality and reliability of tumour molecular profiling in clinical practice [4, 29]. Manual cell counting could be a more accurate methodology but is not routinely carried out in diagnostic practice due to prohibitive demands on time [28]. DL algorithms for automated TPE address the need for higher robustness, reproducibility, and standardisation at low cost [3, 32]. Computer‐aided diagnostic decisions can offer valuable support to pathologists in clinical practice as described elsewhere [33, 34].

We distinguish DL‐based approaches to TPE into (1) WSI‐level methods, where TP is directly predicted as a WSI‐level score, (2) tile‐level methods, where WSIs are tessellated and each tile is classified into tumour and non‐tumour or TP is directly predicted for each tile, and (3) cell‐level methods, where cells are detected and classified into tumour and non‐tumour cells. Supplementary material, Table S2 provides an overview from the literature for each approach. From approaches 1–3 we note an increase in detail and interpretability. From a pathology standpoint, the cell counting methodology represents the most direct measurement of tumour DNA content and is robust with regard to cellular compression, so it is our chosen methodology. Several applications split this task into tissue and cell segmentation, with cells classified into tumour and non‐tumour classes based on their localisation within a predicted tissue compartment, but this is not an exact representation of biological reality. Many published methods were developed for a specific cancer indication and lacked broad applicability across different cancer types. Further, accessibility is limited, with seven out of ten considered cell‐level methods not available for public access and one requiring further fine‐tuning by experts (supplementary material, Table S2). Lastly, validation and comparison against other state‐of‐the‐art approaches are often incomplete. Our chosen method, SoftCTM [16], addresses these concerns. SoftCTM is a multiorgan, open‐source (https://github.com/lely475/ocelot23algo, last accessed: 27 May 2024) solution for detecting tumour and non‐tumour cells, unrestricted by tissue segmentation prediction, developed as part of the OCELOT challenge (https://ocelot2023.grand-challenge.org, last accessed: 27 May 2024). It achieved third place in mean F1 on the OCELOT multiorgan test set [17] compared to 14 other methods, with a difference of only 0.69% mean F1 to the first place method (Test Leaderboard: https://ocelot2023.grand-challenge.org/evaluation/test/leaderboard/, last accessed: 27 May, 2024).

Here, we apply SoftCTM on three large and diverse CRC cohorts, consisting of a randomised clinical trial for patients with advanced disease [11], the CRC TCGA dataset, and a cohort of preoperative rectal biopsies, for a total of over 1,000 samples, including serial sections taken in alternation with tissue material sampled for RNA and DNA profiling within the S:CORT programme. This design ensures continuity of tissue sections extracted for molecular and imaging purposes and is a unique setting to compare the accuracy of CPATH and genomic methods for the determination of TPE within the same sample. For all samples, SoftCTM TPEs were compared with three established approaches (CP, ESTIMATE, InfiniumPurify) widely used in both research and clinical workflows. TPEs by these three methods showed very different distributions. Specifically, the average TPE by CP stood at 35.88% (SD ±13.11), contrasting sharply with InfiniumPurify at 62.66% (SD ±11.82), and even more so with ESTIMATE at 79.24% (SD ±10.48). However, these variations correspond to underlying biology as they all show expected associations with CMS classification. Notably, SoftCTM presents a middle ground, with a mean of 58.87 and broader distribution (SD ±16.32), indicative of potentially more accurate estimations due to its direct cell count measurement. Overestimation can occur in ESTIMATE due to its exclusive measurement of stromal and immune components as the non‐tumour fraction, neglecting the contribution of other normal cell types. In InfiniumPurify, normal tissue, and tumour cells are distinguished, but methylation specific to stromal/immune cells is overlooked. For both methods, this leads to overestimation of tumour cell content, particularly in cases of low purity where the impact of uncaptured non‐tumour biology is strongest. Further, underestimation of TP by CP compared to CPATH or molecular methods is uncommon, as many studies report an overestimation [33, 35, 36, 37, 38, 39]. We speculate that this discrepancy may stem from visual pathologist assessment influenced by variability in tissue appearance and cell compression across different cancer types and pathology workflows. Additionally, pathologist training plays a role, with some sources documenting both over‐ and underestimations [19, 29, 30, 40]. This underscores the necessity for more standardised and strictly quantitative methodologies.

As a result of the TPE underestimation by CP and overestimation by deconvolution methods, the adjusted arm‐level copy numbers result in consistent overcalls and undercalls by around 10% respectively compared to SoftCTM. In contrast, comparison between paired H&Es with SoftCTM shows only ~1.5% differing calls, considered to be expectable background noise. Hence, the 10% differing calls for intermethod comparison contrasted by 1.5% for intra‐method variability highlight the impact of TPE methods on subsequent molecular analysis. This is consistent with and expands recent observations by others [31]. Although the overall patterns of genomic profiles may not be strongly biased, this level of difference could have a tangible effect on future precision medicine pipelines and impact clinical decisions. In addition, deconvolution methods may be confounded by some assumptions such as level of molecular intra‐heterogeneity and/or ploidy, where near‐diploid or near‐tetraploid may provide similar genomic patterns. An accurate, unbiased cell count of tumour and non‐tumour cells before DNA/RNA extraction may provide more objective TPEs to consider for further downstream analyses.

The strengths of this study lie in its comprehensive comparison of TPE assessments across relevant clinical settings, including both resection and biopsy samples. The reference method SoftCTM is publicly available, fully reproducible, and robust with regards to slide selection. SoftCTM predictions are interpretable, as cell markups can be generated for pathologist review (supplementary material, Figure S2). This highlights the applicability and usability of CPATH for TPE. SoftCTM was not initially trained on CRC histology, but it still achieves high agreement of TPEs with a pathologist‐supervised CPATH algorithm for CRC trained on the test cohorts. Still further validation of SoftCTM with regards to cell detection performance for CRC and other indications beyond the OCELOT dataset is recommended.

Overall, SoftCTM showed excellent consistency across slides, was biologically sound, and showed reliable estimates of TPE that were directly interpretable by pathologists. Subsequent steps could lead to the development of image‐based methods for tumour diagnosis together with sample selection and downstream bioinformatic pipelines in research and clinical labs for accurate molecular profiling. While ambitious, recent successes in the classification of cancers at the intersection of digital and molecular pathology make this a plausible next development step [41].

## Author contributions statement

ED, TM, J Rittscher and VHK jointly conceived the study. ED, TM, J Rittscher and VHK designed the study. ED, LAS, TM, J Rittscher and VHK drafted the manuscript. ED, KS, SR, KR, ABl, J Rittscher, CH, CW, IT, ABe, UMcD, GIM, LMS, MS, PQ, TM and VHK obtained and categorised image data, clinicopathological and molecular data. LAS designed and implemented the DP algorithm and performed the DP analysis. ED, LAS, KS, J Rittscher, TM and VHK performed data interpretation. KDM, CV and SL provided important intellectual input, provided critical resources or funding and critically reviewed the study design. ED, LAS, AC, KS, J Robineau and ABl performed bioinformatic and statistical analysis. All authors have read and given approval of the final manuscript.

## Supporting information


**Figure S1.** Overall and cohort‐specific overlap of available data and resources
**Figure S2.** Visualisation of the SoftCTM predictions for areas from two randomly selected WSIs for each cohort
**Figure S3.** Correlation of TPE by SoftCTM and HALO DP, a cell detection algorithm developed within the digital image analysis platform HALO AI™ by IndicaLabs
**Figure S4.** Scatter plots comparing SoftCTM TPE, tumour and background cell counts for H&E section 1 and 2 in FOCUS (*N* = 334), GRAMPIAN (*N* = 149) and both combined (*N* = 483), to assess reproducibility and dependency of SoftCTM on the selected tissue section (all *p* < 0.001)
**Figure S5.** Scatter plot showing the total number of cells for each cohort over the ROI
**Figure S6.** Bland–Altman plots comparing results for each TPE method, bias is indicated as a thick black line, the levels of agreement as dotted lines (all *p* < 0.001)
**Figure S7.** Boxplot comparing distribution of TP estimated by different method for each test cohort
**Figure S8.** Box plots for TPE by different methods for each CMS subtype
**Figure S9.** Comparison of TPE method results for (A) FOCUS, (B) TCGA and (C) GRAMPIAN cohorts
**Table S1.** List of all S:CORT case ids used in this study and their cohort origin (FOCUS or GRAMPIAN)
**Table S2.** Selected examples of automated TPE methods

## Data Availability

FOCUS raw expression data and molecular metadata are publicly available at GEO under reference GSE156915. The transcriptomic data from GRAMPIAN are publicly available at the following link: https://www.scort.org/sites/default/files/exports/scort_ws3_grampian_export_84m9fndk/ws3_grampian_expression_raw.zip. Sequencing data from whole S:CORT are publicly available in EGA (EGAS00001001521). Additional S:CORT data are available to all academic researchers on submission of a data request to the data access committee. For commercial agencies, the data will be made available through Cancer Research Horizons acting on behalf of the funders and consortium members. The TCGA datasets and images analysed in this study are openly and publicly available at https://portal.gdc.cancer.gov/.

## Appendix A

**S:CORT consortium**

**Table:**

**Name**	**Affiliation**
Richard Adams	University of Cardiff
Michael Youdell	University of Oxford
Viktor Koelzer	University of Oxford
Simon Bach	University of Birmingham
Andrew Beggs	University of Birmingham
Celina Whalley	University of Birmingham
Louise Brown	University College London
Francesca Buffa	University of Oxford
Peter Campbell	Wellcome Trust Sanger Institute
Jean‐Baptiste Cazier	University of Birmingham
Enric Domingo	University of Oxford
Andrew Blake	University of Oxford
Chieh‐His Wu	University of Southampton
Aikaterini Chatzipli	Wellcome Trust Sanger Institute
Claire Hardy	Wellcome Trust Sanger Institute
Susan Richman	University of Leeds
Philip Dunne	Queens University Belfast
Keara Redmond	Queens University Belfast
Paul Harkin	Almac Diagnostics
Steven Walker	Almac Diagnostics
Geoff Higgins	University of Oxford
Jim Hill	Christie Hospital Manchester
Chris Holmes	University of Oxford
Denis Horgan	European Association of Precision Medicine
Rick Kaplan	University College London
Richard Kennedy	Queens University Belfast
Mark Lawler	Queens University Belfast
Simon Leedham	University of Oxford
Tim Maughan	University of Oxford
Ultan McDermott	AstraZeneca
Gillies McKenna	University of Oxford
Gary Middleton	University of Birmingham
Dion Morton	University of Birmingham
Graeme Murray	University of Aberdeen
Phil Quirke	University of Leeds
Sanjay Rathee	University of Cambridge
James Robineau	University of Oxford
Manuel Salto‐Tellez	Queens University Belfast
Les Samuel	Grampian NHS Health Board
Anna Schuh	University of Oxford
David Sebag‐Montefiore	University of Leeds
Matt Seymour	University of Leeds
Ricky Sharma	Varian Medical Systems
Richard Sullivan	Kings College London
Ian Tomlinson	University of Oxford
Nicholas West	University of Leeds
Richard Wilson	University of Glasgow
