# Supplementary material for: Tumour purity assessment with deep learning in colorectal cancer and impact on molecular analysis
Source: J Pathol. 2024 Dec 22;265(2):184–97. doi: 10.1002/path.6376 (PMC11717495; doi:10.1002/path.6376)
Supplement: Supplementary file 1 — Figure S1. Overall and cohort‐specific overlap of available data and resources Figure S2. Visualisation of the SoftCTM predictions for areas from two randomly selected WSIs for each cohort Figure S3. Correlation of TPE by SoftCTM and HALO DP, a cell detection algorithm developed within the digital image analysis platform HALO AI™ by IndicaLabs Figure S4. Scatter plots comparing SoftCTM TPE, tumour and background cell counts for H&E section 1 and 2 in FOCUS (N = 334), GRAMPIAN (N = 149) and both combined (N = 483), to assess reproducibility and dependency of SoftCTM on the selected tissue section (all p < 0.001) Figure S5. Scatter plot showing the total number of cells for each cohort over the ROI Figure S6. Bland–Altman plots comparing results for each TPE method, bias is indicated as a thick black line, the levels of agreement as dotted lines (all p < 0.001) Figure S7. Boxplot comparing distribution of TP estimated by different method for each test cohort Figure S8. Box plots for TPE by different methods for each CMS subtype Figure S9. Comparison of TPE method results for (A) FOCUS, (B) TCGA and (C) GRAMPIAN cohorts Table S1. List of all S:CORT case ids used in this study and their cohort origin (FOCUS or GRAMPIAN) Table S2. Selected examples of automated TPE methods [file PATH-265-184-s001.zip › path6376-sup-0001-FiguresS1-S9,TableS2.docx]

**Tumour purity assessment with deep learning in colorectal cancer and impact on molecular analysis**

LA Schönpflug, A Chatzipli *et al. J Pathol* <https://doi.org/10.1002/path.6376>

**Supplementary Figures S1-S9**

**Supplementary Tables S1-S2**

**Figure S1.** Overall and cohort-specific overlap of available data and resources. The histogram bars represent the number of samples with all specified data types available. (A) All, (B) FOCUS, (C) TCGA, and (D) GRAMPIAN. SoftCTM and CP refer to TP estimated by the respective method, H&E1 and H&E2 refer to the availability of two serial H&E-stained sections for (digital) pathology evaluation and transcriptome, NGS, and methylation for the available molecular profiling data of the samples.

**Figure S2.** Visualisation of SoftCTM predictions for areas from two randomly selected WSIs for each cohort. (A) FOCUS, (B) TCGA, and (C) GRAMPIAN. Neoplastic cell detections are marked in blue, non-neoplastic in yellow.

**Figure S3.** Correlation of TPE by SoftCTM and HALO DP, a cell detection algorithm developed within the digital image analysis platform HALO AI^TM^ by IndicaLabs. (A) All cohorts, (B) FOCUS, (C) TCGA, and (D) GRAMPIAN cohorts. Below diagonal: Scatter plots comparing SoftCTM and HALO DP TPE results; diagonal: histogram for SoftCTM and HALO DP with mean (M) and standard deviation (SD) in top right; above diagonal: Pearson correlation coefficient between SoftCTM and HALO DP. *****p* < 0.0001, ***0.001 ≤ *p* ≤ 0.0001, **0.01 ≤ *p* ≤ 0.001, *0.05 ≤ *p* ≤ 0.01, ns: *p* > 0.05. CI, confidence interval.

**Figure S4.** Scatter plots comparing SoftCTM TPE, tumour, and background cell counts for H&E sections 1 and 2 in FOCUS (*N* = 334), GRAMPIAN (*N* = 149), and both combined (*N* = 483) to assess reproducibility and dependency of SoftCTM on selected tissue section (all *p* < 0.001).

**Figure S5.** Scatter plot showing total number of cells for each cohort over region of interest (ROI). We considered only tissue areas for ROI calculation; whitespace areas were excluded. *p* < 0.001.

**Figure S6.** Bland–Altman plots comparing results for each TPE method; bias is indicated as thick black line, levels of agreement as dotted lines (all *p* < 0.001).

**Figure S7.** Boxplot comparing distribution of TP estimated by different methods for each test cohort. (A) FOCUS, (B) TCGA, and (C) GRAMPIAN. We considered only samples with TPE available for all methods. The box represents the interquartile range (IQR), encompassing 50% of the data points. The red line indicates the median, and the whiskers extend to ±1.5 IQR from the xIQR edges.

**Figure S8.** Box plots for TPE by different methods for each CMS subtype. (A) SoftCTM, (B) CP, (C) ESTIMATE, and (D) InfiniumPurify.

**Figure S9.** Comparison of TPE method results for (A) FOCUS, (B) TCGA, and (C) GRAMPIAN cohorts. Below diagonal: scatter plots comparing respective TPE method results. Diagonal: histogram for each TPE method with mean (M) and standard deviation (SD) in top right. Above diagonal: Pearson correlation coefficient between respective TPE methods; significance: *****p* < 0.0001, ***0.001 ≤ *p* ≤ 0.0001, **0.01 ≤ *p* ≤ 0.001, *0.05 ≤ *p* ≤ 0.01, ns: *p* >0.05. CI, confidence interval.

# **Supplementary Tables** **S1-S2**

**Table S1 is provided as a separate Excel file due to its length**

**Table S2**. Selected examples of automated TPE methods.

| **Method** | **TPE level** | **Year** | **Open source?** | **Requires fine-tuning?** | **Cell classification by tissue segmentation?** | **Considered organs** |
| --- | --- | --- | --- | --- | --- | --- |
| [34] | WSI | 2022 | Yes [49] | No | N/A | 10 |
| [42] | WSI | 2022 | Yes [50] | No | N/A | 6 (adrenal gland, lung, breast, head and neck, ovarian, prostate) |
| HistCNN [39] | Tile | 2020 | Yes [51] | No | N/A | 19 |
| [43] | Tile | 2021 | No | N/A | N/A | 1 (breast) |
| HEMnet [44] | Tile | 2022 | Yes [52] | No | N/A | N/A |
| [3] | Cell | 2015 | No | N/A | No | 5 (lung, colon, breast, prostate, ovarian) |
| [45] | Cell | 2017 | No | N/A | No | 1 (breast) |
| [18] | Cell | 2019 | No | N/A | Yes | 1 (CRC) |
| HoverNet [46] | Cell | 2019 | Yes [53] | No | No | 19 |
| [31] | Cell | 2021 | No | N/A | Yes | 1 (lung) |
| [47] | Cell | 2021 | No | N/A | Yes | 3 (CRC, lung, breast) |
| [48] | Cell | 2023 | No | N/A | No | 1 (lung) |
| [33] | Cell | 2023 | No | N/A | Yes | 1 (CRC) |
| QuANTUM [30] | Cell | 2024 | Yes [54] | Yes | No | 1 (lung) |
| SoftCTM [16] | Cell | 2024 | Yes [55] | No | No | 6 (bladder, endometrium, head and neck, kidney, prostate, and stomach) |

N/A indicates category is not applicable or unpublished.
